# Supplementary material for: The association between perceived stress with sleep quality, insomnia, anxiety and depression in kidney transplant recipients during Covid-19 pandemic
Source: PLoS One. 2021 Mar 8;16(3):e0248117. doi: 10.1371/journal.pone.0248117 (PMC7939354; doi:10.1371/journal.pone.0248117)
Supplement: S1 File — (DOCX) [file pone.0248117.s001.docx]

**SOSYODEMOGRAFİK FORM**

1. Cinsiyet: Kadın Erkek
2. Yaş: Boy: Kilo:
3. Medeni durum: Bekar Evli Dul Boşanmış
4. Eğitim Durumu: Okur-Yazar değil İlköğretim Lise Üniversite
5. Sosyo-ekonomik düzey: Düşük Orta Yüksek
6. Herhangi bir işte çalışıyor musunuz? Evet Hayır
7. Kimlerle yaşıyorsunuz? Yalnız Çekirdek aile Geniş aile
8. Böbrek nakli olmadan önce kaç yıl böbrek hastalığı ile takip edildiniz?
9. Böbrek yetmezliği nedeni neydi? Şeker hastalığı Yüksek tansiyon Böbrek taşı Nefrit Polikistik böbrek Diğer
10. Kaç yıldır böbrek nakli ile takip ediliyorsunuz ?
11. **Bu ilk böbrek nakliniz mi?**  Hayır Evet
12. **Hiç rejeksiyon (böbrek reddi) atağı geçirdiniz mi?**  Hayır Evet
13. Genel psikolojik sağlık:

Sürekli tedavi almamı gerektiren süreğen bir psikiyatrik hastalığım yok

Sürekli tedavi almamı gerektiren süreğen bir psikiyatrik hastalığım var

1. Sigara kullanımı: Yok Var
2. Alkol kullanımı: Yok Var
3. Covid-19 enfeksiyonunu……. Geçirmedim

Hafif/orta semptomlar ile geçirdim

Ağır semptomlar ile geçirdim

1. Ailede, yakın çevrede Covid-19 geçirmiş olan…….. Yok Var
2. Ailede, yakın çevreden birinin, Covid-19 nedeni ile ağırlaşarak yoğun bakımda yatışı veya ölümü……. Yok Var
3. Salgın süresince hastaneye ulaşmakta zorluk çektiniz mi? Hayır Evet
