# Supplementary material for: The association between perceived stress with sleep quality, insomnia, anxiety and depression in kidney transplant recipients during Covid-19 pandemic
Source: PLoS One. 2021 Mar 8;16(3):e0248117. doi: 10.1371/journal.pone.0248117 (PMC7939354; doi:10.1371/journal.pone.0248117)
Supplement: S3 File — (PDF) [file pone.0248117.s003.pdf]

Insomnia Severity Index

The Insomnia Severity Index has seven questions. The seven answers are added up to get a total score. When you have your total score, look at the 'Guidelines for Scoring/Interpretation' below to see where your sleep difficulty fits.

For each question, please CIRCLE the number that best describes your answer.

Please rate the CURRENT (i.e. LAST 2 WEEKS) SEVERITY of your insomnia problem(s).

Insomnia Problem	None	Mild	Moderate	Severe	Very Severe
1. Difficulty falling asleep	0	1	2	3	4
2. Difficulty staying asleep	0	1	2	3	4
3. Problems waking up too early	0	1	2	3	4

4. How SATISFIED/DISSATISFIED are you with your CURRENT sleep pattern?

Very Satisfied	Satisfied	Moderately Satisfied	Dissatisfied	Very Dissatisfied
0	1	2	3	4

5. How NOTICEABLE to others do you think your sleep problem is in terms of impairing the quality of your life?

Not at all Noticeable	A Little	Somewhat	Much	Very Much Noticeable
0	1	2	3	4

6. How WORRIED/DISTRESSED are you about your current sleep problem?

Not at all Worried	A Little	Somewhat	Much	Very Much Worried
0	1	2	3	4

7. To what extent do you consider your sleep problem to INTERFERE with your daily functioning (e.g. daytime fatigue, mood, ability to function at work/daily chores, concentration, memory, mood, etc.) CURRENTLY?

Not at all Interfering	A Little	Somewhat	Much	Very Much Interfering
0	1	2	3	4

Guidelines for Scoring/Interpretation:

Add the scores for all seven items (questions 1 + 2 + 3 + 4 + 5 +6 + 7) = _______ your total score

Total score categories:
0–7 = No clinically significant insomnia
8–14 = Subthreshold insomnia
15–21 = Clinical insomnia (moderate severity)
22–28 = Clinical insomnia (severe)

Used via courtesy of www.myhealth.va.gov with permission from Charles M. Morin, Ph.D., Université Laval
