# Supplementary material for: The association between perceived stress with sleep quality, insomnia, anxiety and depression in kidney transplant recipients during Covid-19 pandemic
Source: PLoS One. 2021 Mar 8;16(3):e0248117. doi: 10.1371/journal.pone.0248117 (PMC7939354; doi:10.1371/journal.pone.0248117)
Supplement: S4 File — (PDF) [file pone.0248117.s004.pdf]

PITTSBURGH SLEEP QUALITY INDEX (PSQI)

INSTRUCTIONS: The following questions relate to your usual sleep habits during the past month only. Your answers should indicate the most accurate reply for the majority of days and nights in the past month. Please answer all questions.

1. During the past month, when have you usually gone to bed at night?

   USUAL BED TIME ______________________

2. During the past month, how long (in minutes) has it usually take you to fall asleep each night?

   NUMBER OF MINUTES ______________________

3. During the past month, when have you usually gotten up in the morning?

   USUAL GETTING UP TIME ______________________

4. During the past month, how many hours of actual sleep did you get at night? (This may be different than the number of hours you spend in bed.)

   HOURS OF SLEEP PER NIGHT ______________________

INSTRUCTIONS: For each of the remaining questions, check the one best response. Please answer all questions.

5. During the past month, how often have you had trouble sleeping because you...

	Not during the past month	Less than once a week	Once or twice a week	Three or more times a week
(a) ...cannot get to sleep within 30 minutes	☐	☐	☐	☐
(b) ...wake up in the middle of the night or early morning	☐	☐	☐	☐
(c) ...have to get up to use the bathroom	☐	☐	☐	☐
(d ...cannot breathe comfortably	☐	☐	☐	☐
(e) ...cough or snore loudly	☐	☐	☐	☐
(f) ...feel too cold	☐	☐	☐	☐
(g) ...feel too hot	☐	☐	☐	☐
(h) ...had bad dreams	☐	☐	☐	☐
(i) ...have pain	☐	☐	☐	☐

(j) Other reason(s), please describe

____

____

	Not during the past month	Less than once a week	Once or twice a week	Three or more times a week
How often during the past month have you had trouble sleeping because of this?	☐	☐	☐	☐

	Very good	Fairly good	Fairly bad	very bad
6. During the past month, how would you rate your sleep quality overall?	☐	☐	☐	☐

	Not during the past month	Less than once a week	Once or twice a week	Three or more times a week
7. During the past month, how often have you taken medicine (prescribed or "over the counter") to help you sleep?	☐	☐	☐	☐
8. During the past month, how often have you had trouble staying awake while driving, eating meals, or engaging in social activity?	☐	☐	☐	☐

	No problem at all	Only a very slight problem	Somewhat of a problem	A very big problem
9. During the past month, how much of a problem has it been for you to keep up enough enthusiasm to get things done?	☐	☐	☐	☐

	No bed partner or roommate	Partner/ roommate in other room	Partner in same room, but not same bed	Partner in same bed
10. During the past month, how much of a problem has it been for you to keep up enough enthusiasm to get things done?	☐	☐	☐	☐

If you have a roommate or bed partner, ask him/her how often in the past month you have had...

	Not during the past month	Less than once a week	Once or twice a week	Three or more times a week
(a) ...loud snoring	☐	☐	☐	☐
(b) ...long pauses between breaths while asleep	☐	☐	☐	☐
(c) ...legs twitching or jerking while you sleep	☐	☐	☐	☐
(d) ...episodes of disorientation or confusion during sleep	☐	☐	☐	☐
(e) Other restlessness while you sleep; please describe	☐	☐	☐	☐

____

____

SCORING INSTRUCTIONS FOR THE PITTSBURGH SLEEP QUALITY INDEX:

The Pittsburgh Sleep Quality Index (PSQI) contains 19 self-rated questions and 5 questions rated by the bed partner or roommate (if one is available). Only self-rated questions are included in the scoring. The 19 self-rated items are combined to form seven "component" scores, each of which has a range of 0-3 points. In all cases, a score of "0" indicates no difficulty, while a score of "3" indicates severe difficulty. The seven component scores are then added to yield one "global" score, with a range of 0-21 points, "0" indicating no difficulty and "21 " indicating severe difficulties in all areas.

Scoring proceeds as follows:

Component 1: Subjective sleep quality

Examine question #6, and assign scores as follows:

Response	Component 1 score
"Very good"	0
"Fairly good"	1
"Fairly bad"	2
"Very bad"	3

Component 1 score: ________

Component 2: Sleep latency

1. Examine question #2, and assign scores as follows:

Respo\nse	Score
≤15 minutes	0
16-30 minutes	1
31-60 minutes	2
> 60 minutes	3
Question #2 score:	________

2. Examine question #5a, and assign scores as follows:

Response	Score
Not during the past month	0
Less than once a week	1
Once or twice a week	2
Three or more times a week	3
Question #5a score:	________

3. Add #2 score and #5a score

Sum of #2 and #5a: ________

4. Assign component 2 score as follows:

Sum of #2 and #5a	Component 2 score
0	0
1-2	1
3-4	2
5-6	3

Component 2 score: ________

Component 3: Sleep duration

Examine question #4, and assign scores as follows:

Response	Component 3 score
> 7 hours	0
6-7 hours	1
5-6 hours	2
< 5 hours	3

Component 3 score:_________

Component 4: Habitual sleep efficiency

1. Write the number of hours slept (question #4) here:_________
2. Calculate the number of hours spent in bed:

    Getting up time (question #3):_________

    Bedtime (question #1):_________

    ____

    *Number of hours spent in bed:*_________

3. Calculate habitual sleep efficiency as follows:

    (Number of hours slept/Number of hours spent in bed) X 100 = Habitual sleep efficiency (%)

    (_________/_________) X 100 = %

4. Assign component 4 score as follows:

Habitual sleep efficiency %	Component 4 score
> 85%	0
75-84%	1
65-74%	2
< 65%	3

Component 4 score:_________

Component 5: Step disturbances

1. Examine questions #5b-5j, and assign scores for each question as follows:

Response	Score
Not during the past month	0
Less than once a week	1
Once or twice a week	2
Three or more times a week	3

5b score: ________

5c score: ________

5d score: ________

5e score: ________

5f score: ________

5g score: ________

5h score: ________

5i score: ________

5j score: ________

2. Add the scores for questions #5b-5j:

Sum of #5b-5j: ________

3. Assign component 5 score as follows:

Sum of #5b-5j	Component 5 score
0	0
1-9	1
10-18-4	2
19-27	3

Component 5 score: ________

Component 6: Use of sleeping medication

Examine question #7 and assign scores as follows:

Response	Component 6 score
Not during the past month	0
Less than once a week	1
Once or twice a week	2
Three or more times a week	3

Component 6 score: ________

Component 7: Daytime dysfunction

1. Examine question #8, and assign scores as follows:

Response	Score
Never	0
Once or twice	1
Once or twice each week	2
Three or more times each week	3

Question#8 score: ________

2. Examine question #9, and assign scores as follows:

Response	Score
No problem at all	0
Only a very slight problem	1
Somewhat of a problem	2
A very big problem	3

Question #9 score: ________

3. Add the scores for question #8 and #9:

Sum of #8 and #9: ________

4. Assign component 7 score as follows:

Sum of #8 and #9	Component 7 score
0	0
1-2	1
3-4	2
5-6	3

Component 7 score: ________

Global PSQI Score

Add the seven component scores together:

Global PSQI Score: ________
