# Supplementary material for: The association between perceived stress with sleep quality, insomnia, anxiety and depression in kidney transplant recipients during Covid-19 pandemic
Source: PLoS One. 2021 Mar 8;16(3):e0248117. doi: 10.1371/journal.pone.0248117 (PMC7939354; doi:10.1371/journal.pone.0248117)
Supplement: S5 File — (PDF) [file pone.0248117.s005.pdf]

The Perceived Stress Scale (14 items) - Cohen et al, 1983

Recommended by The NIH Centers for Population Health and Health Disparities (CPHHD)-Measures and Methods Work Group (MMWG)

CPHHD Taxonomy- Health and Mental Health [Well-being]-stress & hypervigilance-Perceived Stress

Also recommended by MacArthur Foundation (see http://www.macses.ucsf.edu/research/psychosocial/stress.php#perceived)

1. In the last month, how often have you been upset because of something that happened unexpectedly?
2. In the last month, how often have you felt that you were unable to control important things in your life?
3. In the last month, how often have you felt nervous and "stressed"?
4. In the last month, how often have you dealt successfully with irritating life hassles?
5. In the last month, how often have you felt that you were effectively coping with important changes that were occurring in your life?
6. In the last month, how often have you felt confident about your ability to handle your personal problems?
7. In the last month, how often have you felt that things were going your way?
8. In the last month, how often have you found that you could not cope with all the things that you had to do?
9. In the last month, how often have you been able to control irritations in your life?
10. In the last month, how often have you felt that you were on top of things?
11. In the last month, how often have you been angered because of things that happened that were outside of your control?
12. In the last month, how often have you found yourself thinking about things that you have to accomplish?
13. In the last month, how often have you been able to control the way you spend your time?
14. In the last month, how often have you felt difficulties were piling up so high that you could not overcome them?

[0=never; 1=almost never; 2=sometimes; 3=fairly often; 4=very often]

Note: Items 4, 5, 6, 7, 9, 10, and 13 are scored in reverse direction.
